# Sensitivity of the reference evapotranspiration to key climatic variables during the growing season in the Ejina oasis northwest China

**DOI:** 10.1186/2193-1801-2-S1-S4

**Published:** 2013-12-11

**Authors:** Lan-gong Hou, Song-bing Zou, Hong-lang Xiao, Yong-gang Yang

**Affiliations:** Geographic Information and Tourism Department, Chuzhou University, Chuzhou, China; Key Laboratory of Eco-hydrology and River Basin Science, Cold and Arid Regions Environmental and Engineering Research Institute, Chinese Academy of Sciences, No. 260 West Donggang Road, Lanzhou, China

**Keywords:** Reference evapotranspiration, FAO Penman-Monteith model, Sensitivity coefficient, Ejina oasis, China

## Abstract

The standardized FAO56 Penman-Monteith model, which has been the most reasonable method in both humid and arid climatic conditions, provides reference evapotranspiration (ETo) estimates for planning and efficient use of agricultural water resources. And sensitivity analysis is important in understanding the relative importance of climatic variables to the variation of reference evapotranspiration. In this study, a non-dimensional relative sensitivity coefficient was employed to predict responses of ETo to perturbations of four climatic variables in the Ejina oasis northwest China. A 20-year historical dataset of daily air temperature, wind speed, relative humidity and daily sunshine duration in the Ejina oasis was used in the analysis. Results have shown that daily sensitivity coefficients exhibited large fluctuations during the growing season, and shortwave radiation was the most sensitive variable in general for the Ejina oasis, followed by air temperature, wind speed and relative humidity. According to this study, the response of ETo can be preferably predicted under perturbation of air temperature, wind speed, relative humidity and shortwave radiation by their sensitivity coefficients.

## Introduction

The evapotranspiration from a reference surface, not short of water, is called the reference evapotranspiration and is denoted as ETo. The reference surface is a hypothetical green grass reference crop of uniform height, actively growing. Being an important component of the hydrological cycle, ETo will affect agricultural water use [1, 2], ecosystem models [3], aridity/humidity conditions [4], and rainfall-runoff estimation. ETo is a measurement of the evaporative demand of the atmosphere independent of crop type, crop development and management practices. Only climatic factors affect ETo. Consequently, ETo is a function of weather parameters and can be computed from meteorological data [5]. Numerous methods have been used to estimate ETo, including: (1) water budget [6], (2) mass-transfer [7], (3) combination [8], (4) radiation [9], and (5) temperature-based [10, 11] equations. However, it causes confusion as to which method to select for ETo estimation. Therefore, the Food and Agriculture Organization of the United Nations proposed Penman-Monteith model in Irrigation and Drainage Paper No. 56 (hereafter as FAO56-PM) using the hypothesized reference crop (height of 0.12 m, surface resistance of 70 sm^-1^ and albedo of 0.23) as the sole method for determining ETo [5, 12]. The FAO56-PM model, which incorporates thermodynamic and aerodynamic aspects, has proved to be a relatively accurate method in both humid and arid climates. And the model has received favorable acceptance and application over much of the world [13–17].

A major drawback to apply the FAO56-PM model is its relatively high data demand. The model requires air temperature, wind speed, relative humidity, and shortwave radiation data. The number of meteorological stations where all of these parameters are observed is limited in many areas of the globe. The number of stations where reliable data for these parameters exist is even smaller, especially in developing countries [18]. A sensitivity analysis of ETo to perturbations (all sorts of data errors or, actual climatic changes) associated with one or more climatic variables is important to improve our understanding of the connections between climatic conditions and ETo variability, and between data availability and estimation accuracy of ETo.

Studies on regional and temporal behavior of the sensitivity of reference evapotranspiration to climatic variables are rare in the literature [19], and so far, no study has been done for the Ejina oasis northwest China. A recent study of the sensitivity of ETo was reported by Hupet and Vanclooster in a moderate humid climatic zone in Belgium [20]. Because of different approaches used in parameterising ET models, there are different definitions of the sensitivity coefficients in previous studies [21–25], which makes it difficult to compare literature results. Thus, a common framework for sensitivity analysis of ETo with long-term dataset would be useful in connecting the temporal variability of sensitivity with regional climate conditions. The aim of the present study was to (1) estimate mean daily reference evapotranspiration during the growing season in the Ejina oasis over the period 1988-2007; (2) provide reliable sensitivity coefficients of ETo for the Ejina oasis northwest China based on meteorological data of Ejina meteorological observatory station over the period 1988-2007. And quantitative estimation of the effect of different meteorological variables on reference evapotranspiration is an important step in studying the impact of climate change on evapotranspiration and water-balance components.

## Materials and methods

### Study area

**Figure 1:**
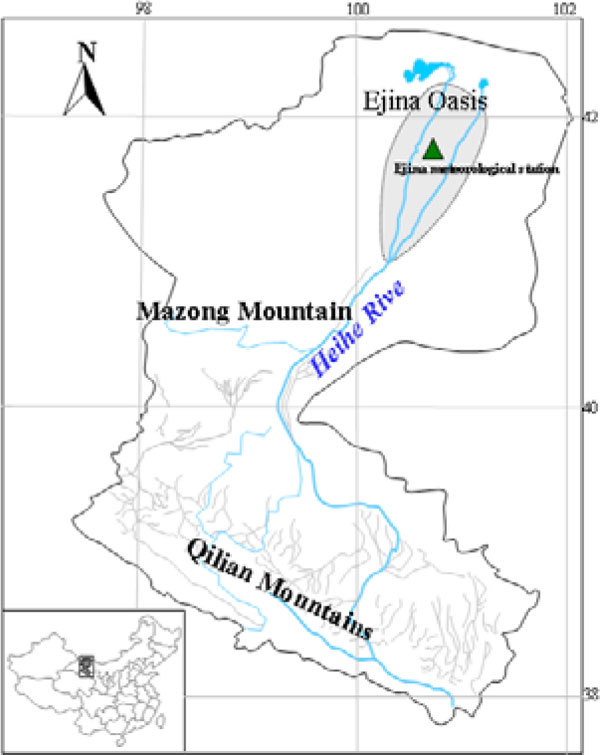
**Sketch of the Heihe river and the study region**.

The Ejina oasis, in the lower reaches of Heihe river, is located in Ejina county, Inner Mongolia, China, and the area is 3328 km^2^ (Figure 1). It is in the hinterland of Asia continent, and is one of the most arid in China. The average annual air temperature is about 6~8.5 °C. The mean annual precipitation, 84% of which occurs during the growing season, is less than 50 mm year^−1^. Prevailing winds are northwesterly in the winter and spring, and southwesterly to southerly in the summer and fall. Annual mean wind velocity ranges from 2.9 to 5.0 m s^−1^.

A data set of Ejina meteorological observatory station with daily observations of maximum, minimum and average air temperature at 2 m height, wind speed measured at 10 m height, relative humidity (2 m height) and daily sunshine duration for the period 1988-2007 was used in this study. Data were provided by the National Climatic Centre (NCC) of China Meteorological Administration (CMA). The wind-speed measurements were transformed to wind speed at 2 m height by the wind profile relationship introduced in Chapter 3 of the FAO paper 56 [5].

### The FAO56 Penman-Monteith equation

The FAO56-PM equation for calculating daily reference evapotranspiration is:

where *ETo* is the reference evapotranspiration (mm day^-1^), *R*_*n*_ the net radiation at the crop surface (MJ m^-2^day^-1^), *G* the soil heat flux density (MJ m^-2^day^-1^), *T* the mean daily air temperature at 2 m height (°C), *u*_*2*_ the wind speed at 2 m height (m s^-1^), *e*_*s*_ the saturation vapor pressure (kPa), *e*_*a*_ the actual vapor pressure (kPa), *e*_*s*_*- e*_*a*_ the saturation vapor pressure deficit (kPa), *Δ* the slope of the vapor pressure curve (kPa °C^-1^) and *γ* is the psychrometric constant (kPa °C^-1^). The computation of all data required for the calculation of the reference evapotranspiration followed the method and procedure given in Chapter 3 of the FAO paper 56 [5].

Original measurements of air temperature (*T*), wind speed (*u*_*2*_), and relative humidity (*RH*) were chosen for sensitivity analyses. The fourth variable that was analyzed is shortwave radiation (*R*_*s*_). This is because shortwave radiation is one of the input variables in a number of semi-physical and semi-empirical equations that are used to derive the net energy flux required by the Penman method. Following the procedure described by Allen et al. [5], *R*_*s*_ can be estimated with the Angstrom formula that relates surface shortwave radiation to extraterrestrial radiation and daily sunshine duration:

where *R*_*S*_ is solar or shortwave radiation (MJ m^-2^day^-1^), *n* is daily sunshine duration (h), *N* is maximum possible duration of sunshine or daylight hours (h), *n/N* is relative sunshine duration, *R*_*a*_ is extraterrestrial radiation (MJ m^-2^day^-1^), *a* and *b* are regression constants. The recommended values *a* = 0.2 and *b* = 0.79 were used in this study [26].

### The sensitivity coefficient

In hydrological studies and ecological applications, a number of sensitivity coefficients have been defined depending on the purpose of the analyses [21, 23, 24, 27, 28]. More often, however, a mathematically defined sensitivity coefficient is used to characterize sensitivity [20–25]. For multi-variable models (e.g., the FAO56-PM model), different variables have different dimensions and different ranges of values, which makes it difficult to compare the sensitivity by partial derivatives. Consequently, the partial derivative is transformed into a non-dimensional form [24]:

Where *S*_*Vi*_ is sensitivity coefficient and Vi is the ith variable. The transformation that gives the ''non-dimensional relative sensitivity coefficient'' (denoted as ''sensitivity coefficient'' in the following text), was first adopted by McCuen and has been now widely used in evapotranspiration studies [19–25]. Basically, a positive/negative sensitivity coefficient of a variable indicates that ETo will increase/decrease as the variable increases. The larger the sensitivity coefficient is, the larger effect a given variable has on ETo. In graphical form, the sensitivity coefficient is the slope of the tangent at the origin of the sensitivity curve. Practically, the coefficient is accurate enough to represent the slope of the sensitivity curve within a certain ''linear range'' around the origin. The width of the range depends on the degree of non-linearity of the sensitivity curve. If a sensitivity curve is linear, the sensitivity coefficient is able to represent the change in ETo caused by any perturbation of the variable concerned.

Sensitivity coefficients were calculated on a daily basis for air temperature, wind speed, relative humidity and shortwave radiation. Average monthly sensitivity coefficients were obtained by averaging daily values.

## Results and discussions

### Climate and daily variation of ETo during the growing season

**Figure 2:**
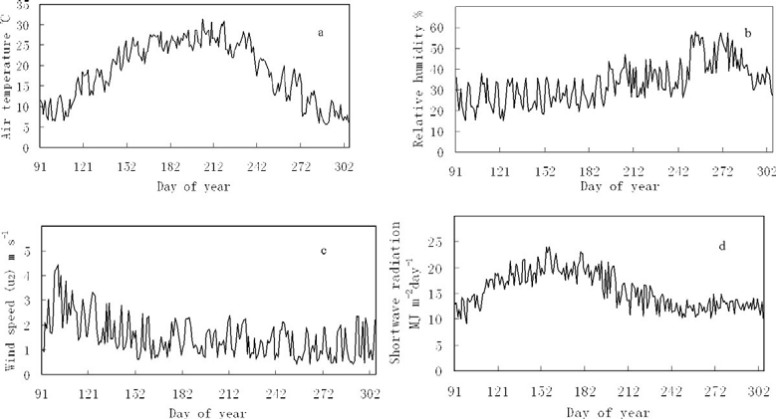
**Mean daily variations of the major climatic variables during the growing season in the Ejina oasis: a) air temperature; b) relative humidity; c) wind speed; d) shortwave radiation**.

In Ejina oasis, climatic variables exhibit large fluctuations during the growing season (Figure 2). Daily variation patterns of air temperature are similar to those of shortwave radiation, and the variation patterns are single-peak. In the early growing season, the mean daily air temperature and shortwave radiation were still low (Figures 2a and 2d). During the middle period of the growing season, air temperature and shortwave radiation reached maximum values, the highest air temperature was in July, and the highest shortwave radiation was in June. Daily variation patterns of relative humidity are opposite to those of wind speed (Figures 2b and 2c). During the growing season, relative humidity increased gradually, and the maximum values were in September and October. But wind speed decreased gradually, and the maximum wind speed occurred in April.

**Figure 3:**
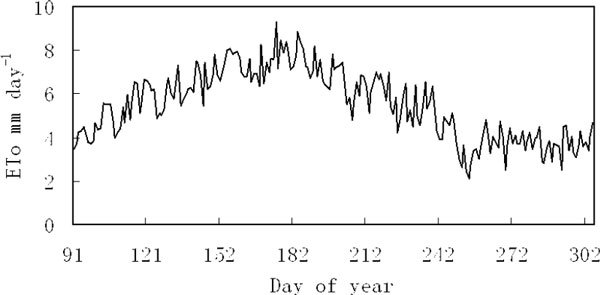
**Mean daily patterns of ETo during the growing season in the Ejina oasis**.

During the growing season, daily variation of ETo fluctuates largely (Figure 3). The daily variation patterns of ETo are single-peak. From the beginning of growing season, the value of ETo increased gradually, and ETo reached the maximum values between June and July. Afterwards, the daily value of ETo decreased gradually.

### Daily variation of the sensitivity coefficients during the growing season

**Figure 4:**
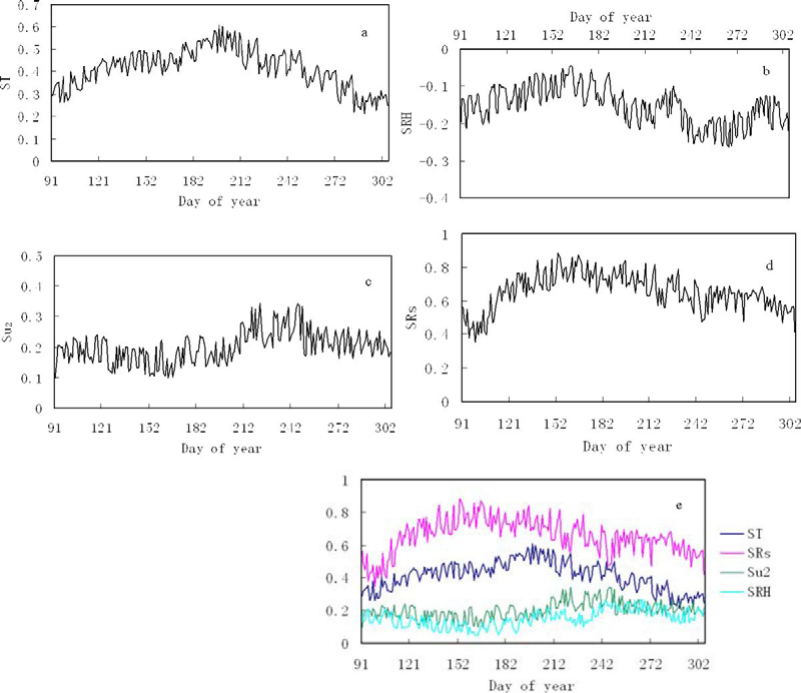
**Mean daily sensitivity coefficients for air temperature (ST) (a), relative humidity (SRH) (b), wind speed (Su**_**2**_ **) (c) and shortwave radiation (SRs) (d) during the growing season in the Ejina oasis**. (e) Comparison of mean daily sensitivity coefficients for major climatic variables in the Ejina oasis (SRH is multiplied by -1 to facilitate visual comparison).

Daily sensitivity coefficients exhibit large fluctuations during the growing season (Figure 4). The same feature has also been reported by Hupet and Vanclooster [20]. Daily variation patterns of ST agree with those of air temperature. ETo was insensitive to air temperature in the early growing season and the sensitivity gradually increased and achieved its maximum value during the middle part of the growing season (June-August) (Figure 4a). The similar patterns of ST and air temperature indicated that air temperature determined the extent of the temporal variation of ST. Negative sensitivity coefficients were obtained for relative humidity (Figure 4b). Negative sensitivity coefficients indicated that increases in relative humidity reduced the evapotranspiration potential. Similar results were obtained in previous studies, where relative humidity was a major limiting factor. Zeng and Heilman concluded that the impact of climate change might be minimal if warming was accompanied by higher humidity [29]. Figure 4c showed that ETo was relative insensitive to wind in the early growing season and during the middle part of the growing season, and more sensitive to wind at the end of the growing season. Daily variation patterns of SRs were similar to those of shortwave radiation. Minimum and maximum values were found in the early growing season and the middle part of the growing season, respectively (Figure 4d). Like air temperature, the sensitivity coefficient for shortwave radiation also showed a pronounced temporal cycle, similar to the temporal cycle of the measured shortwave radiation. A decrease in the energetic term appeared to be associated with an increased significance of the aerodynamic term, which led to the decrease of the sensitivity coefficients for the shortwave radiation corresponded to an increase in the sensitivity coefficient for the wind speed at the end of the growing season. Similar findings were reported elsewhere [20, 22–24]. ST and SRs had a similar pattern while opposite patterns were found for SRH and Su_2_. In general, shortwave radiation was the most sensitive variable at the daily scale, and air temperature was less influential to ETo. According to this study, we found wind speed and relative humidity to be the least sensitive variables in Ejina oasis throughout the growing season, but their sensitivities were opposite to each other. Ejina oasis is in the extreme arid region northwest China, where relative humidity is always relative lower, so there is less impact of relative humidity on ETo. Then daily variation patterns of SRH are different from other study [19].

## Conclusions

Reference evapotranspiration and sensitivities of reference evapotranspiration to four major climatic variables were studied during the growing season in the Ejina oasis northwest China using a 20-year dataset. Daily variation of ETo fluctuates largely, and the daily variation patterns of ETo are single-peak. The values of ETo were low in the early growing season and the values gradually increased and achieved the maximum value during the middle part of the growing season (June-August). The study showed that shortwave radiation was the most sensitive variable in general for the Ejina oasis, followed by air temperature, which had similar variation patterns of sensitivity to those of SRs. Wind speed and relative humidity had the least impact, which had opposite variation patterns of sensitivity.

The results of this work can be used as a theoretical basis for future research on the response of reference evapotranspiration to climatic change. The long-term variability of the sensitivity coefficients indicated that the ETo response to climate change will differ with time. Generally, the non-dimensional relative sensitivity coefficient (*S*_*Vi*_) gave satisfactory prediction of the ETo response to a perturbation of one or more climatic variables.
